# Traitement chirurgical des métastases osseuses rachidiennes

**DOI:** 10.11604/pamj.2017.26.153.8598

**Published:** 2017-03-15

**Authors:** Abderrazzak El Saqui, Mohamed Aggouri, Mohamed Benzagmout, Khalid Chakour, Mohamed El Faiz Chaoui

**Affiliations:** 1 Service Neurochirurgie, CHU Hassan II, Fès, Maroc

**Keywords:** Palliative surgery, osteocondensation metastasis, multidisciplinary staff, Palliative surgery, osteocondensation metastasis, multidisciplinary staff

## Abstract

La chirurgie de la métastase est une chirurgie palliative le plus souvent, surtout au niveau du rachis de par sa localisation anatomique. Cette chirurgie doit être bien préparée pour que les suites soient simples et que le traitement adjuvant soit fait dans les meilleurs délais. La stratégie thérapeutique doit être systématiquement prise en réunion de concertation pluridisciplinaire (RCP). Le principal risque de la localisation rachidienne est neurologique, c'est pourquoi cette chirurgie doit être préventive le plus souvent. Elle a pour objectif principal d'améliorer la qualité de vie.

## Abstract (en)

Surgery for metastatic tumor is usually a palliative surgery, especially for spinal metastases, due to their anatomical localization. Surgical procedure should be accurately established to have simple outcomes and to start adjuvant treatment as soon as clinically possible. Therapeutic strategy should proceed after multidisciplinary consultation meeting (RCP). The main risk of spinal bone metastases is neurological, hence this surgery should be most commonly preventive. Its main objective is to improve patient's quality of life.

## Introduction

La chirurgie des métastases osseuses n'est pas comparable à celle des tumeurs primitives. Elle est le plus souvent palliative, les métastases uniques étant très rares. C'est pourquoi la stratégie chirurgicale doit prendre en compte des paramètres primordiaux tels que le cancer primitif, l'objectif recherché, le stade de la maladie, l'état général du patient. Une réunion multidisciplinaire est donc primordiale avant toute décision chirurgicale. Les métastases rachidiennes sont une entité à part entière, en raison du risque neurologique qu'elles entraînent. Le patient devra donc être bien préparé pour éviter des complications lourdes avant ou après l'opération. Cela conditionne le pronostic, car la chirurgie des métastases osseuses n'est qu'une “parenthèse”dans le traitement global de la maladie. En général, la radiothérapie peut se faire à J + 3 semaines, et la chimiothérapie, si nécessaire, à J +10 jours .La métastase est condensante, parfois lysante ou mixte. Le risque fracturaire est donc moindre, mais il ne faut pas considérer cet os comme solide : il est en effet fragile comme du verre. Au niveau vertébral, la stratégie thérapeutique dépend du niveau rachidien, de l'envahissement de la vertèbre et du stade de la maladie primitive.

## Méthodes

**Figure 1:**
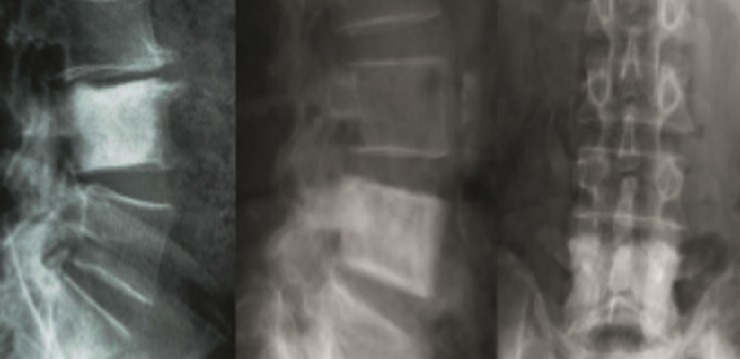
Radiographie standard face et profil du rachis lombaire objectivant une vertèbre L4 en ivoire (hyperdense)

**Figure 2:**
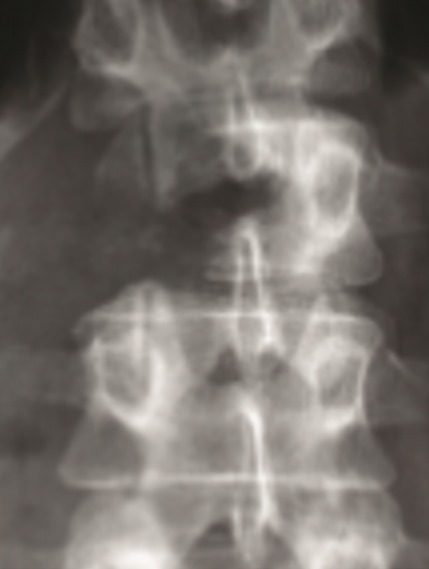
Radiographie standard de face : vertèbre borgne métastatique (le pédicule droit n’existe plus)

**Figure 3:**
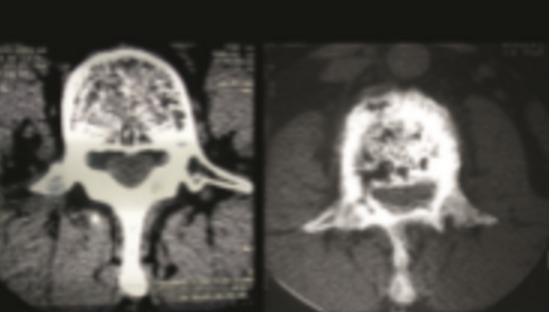
TDM du rachis coupe axiale montrant un envahissement métastatique d’une vertèbre ivoire

**Figure 4:**
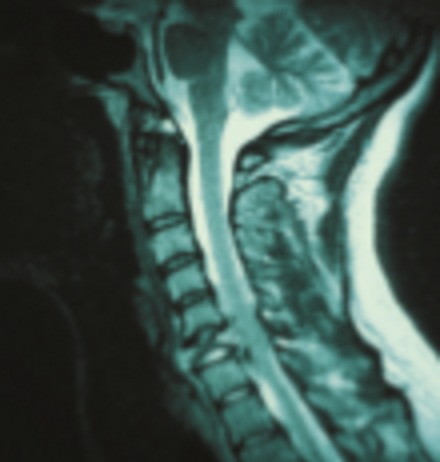
IRM du rachis cervical en coupe sagittale, séquence T2 objectivant une métastase unique de C6

**Stratégie chirurgicale selon la localisation anatomique:** La localisation sur le squelette nécessite une préparation différente à chaque fois. Le petit bassin et le sacrum sont des régions profondes impliquant une chirurgie techniquement difficile. Le bilan local doit toujours être identique et comporter : -Une radiographie standard de face et de profil, avec la fameuse vertèbre ivoire caractéristique de la métastase prostatique; -Une IRM de la région tumorale; -Un scanner de la région tumorale permettant d'apprécier le caractère lytique, condensant ou mixte de la métastase (Figure 1, Figure 2). Il faut préciser que le scanner et l'IRM sont totalement complémentaires pour la bonne compréhension de la tumeur par le chirurgien. L'IRM peut induire en erreur le chirurgien en cas de métastase prostatique, car elle ne permet pas d'affirmer la nature lytique ou condensante de la tumeur. La résolution du scanner est bien supérieure dans ce cas. La radiologie standard est primordiale, car elle permet d'avoir une vue d'ensemble de l'envahissement tumoral durant l'intervention chirurgicale (Figure 3, Figure 4).

**Etat actuel des connaissances:** Le bilan général indiquera le stade de la maladie métastatique, ce qui permettra d'adopter la meilleure stratégie thérapeutique. Il comporte : -Un scanner thoraco-abdomino-pelvien (scan-TAP); -Un TEP scan; -Parfois un scanner cérébral ou une scintigraphie osseuse corps entier. Les préparations particulières pour les lésions situées dans le petit bassin sont une bonne préparation colique, la pose, si nécessaire, d'une sonde en double J du côté lésionnel pour éviter une plaie de l'uretère, et une angiographie pour s'assurer que les vaisseaux ne sont pas envahis. Pour ce type de chirurgie lourde et complexe, l'intervention de plusieurs équipes chirurgicales peut être nécessaire.

**Métastases osseuses rachidiennes:** Les indications et la technique chirurgicale dépendent de l'envahissement sur la vertèbre, du niveau rachidien (cervical, dorsal, lombaire ou sacré), du risque mécanique et neurologique. Il faut savoir que la chirurgie carcinologique réséquant “en bloc”la tumeur est extrêmement rare, en raison de l'anatomie de la vertèbre (proximité du canal rachidien). La plupart du temps, la chirurgie sera palliative, pour éviter un risque mécanique de fracture entraînant un risque neurologique. R. Roy-Camille [1] avait bien décrit les conditions de la faisabilité d'une chirurgie carcinologique ou non. Il se fondait sur l'atteinte de 1 ou des 2 pédicules. Cet envahissement selon le niveau rachidien permettait ou non la résection tumorale “en bloc”. Par la suite, d'autres auteurs, comme Y. Tokuhashi et al. [2], ont élaboré des recommandations pour orienter la technique chirurgicale. E.A. Enkaoua et al. [3] ont émis des critiques concernant ces recommandations et ont élaboré un arbre décisionnel incluant la notion d'envahissement vertébral et le niveau rachidien (score ISBM [International Society of Bone Metastasis]) [4, 5]. Ce score comprend 4 classes. La classe 4 regroupe les métastases non chirurgicales. Les 3 autres sont les suivantes. Classe 1: métastase unique. Elle apparaît 3 ans après la découverte du cancer primitif. Il faut alors traiter cette métastase de façon agressive, en essayant de faire une chirurgie carcinologique, si possible. Classe 2: métastase neurologique. Il s'agit d'une urgence thérapeutique. Cette atteinte peut être partielle ou totale. Il faut agir en général dans les 6 heures suivant le début du déficit, car la compression tumorale est plus vasculaire que mécanique. Cette classe ne concerne que les métastases thoraciques et cervicales. Classe 3: métastases présentant un risque mécanique ou neurologique. Ce sont les cas majoritaires.

L'arbre décisionnel comprend 4 items.

**L'espérance de vie du patient:** Inférieure à 1 an: 1 point; entre 1 et 2 ans: 3 points; supérieure à 2 ans: 6 points. Ainsi, plus l'espérance de vie est élevée, plus la chirurgie est indiquée. Plusieurs éléments peuvent pondérer cet item: un indice de Karnofsky (état général) inférieur à 70 %, la présence de métastases viscérales et la présence de métastases multiples osseuses. Si ces 3 éléments sont présents, le score sera de 1. Si 2 éléments sont présents, le score sera de 3 s'il était de 6 et de 1 sinon.

**La localisation au niveau du rachis:** cervicale: 2 points; thoracique: 3 points; lombaire: 1 point. Plus le risque neurologique est élevé, plus la chirurgie est indiquée.

**La localisation sur la vertèbre:** Selon F. Denis [6], la vertèbre est divisée en 3 parties (antérieure, moyenne et postérieure): si seule 1 partie est envahie: 1 point; si seules 2 parties sont envahies: 2 points; si toute la vertèbre est envahie: 3 points. Ainsi, plus la vertèbre est envahie, plus la chirurgie est indiquée.

**L'efficacité du traitement adjuvant (radiothérapie ou chimiothérapie) :** -S'il existe un traitement efficace : 0 point ; -s'il n'y a pas de traitement adjuvant efficace : 3 points. Ainsi, en reprenant les 4 items avec leurs pondérations: score entre 3 et 6: pas de chirurgie; score entre 7 et 11: chirurgie palliative; score entre 12 et 15: chirurgie agressive.

Il faut savoir que, si l'index de Karnofsky est inférieur à 50 %, le résultat de la chirurgie est en général très aléatoire. Cette chirurgie peut être très hémorragique, surtout en cas de métastase rénale ou thyroïdienne ; une embolisation préopératoire, si elle est possible, est fortement conseillée. Il ne faut pas hésiter à administrer, à la fin de chaque intervention de ce type, des produits hémostasiants pour éviter un hématome rachidien. En effet, au réveil, la tension du patient remonte et peut faire saigner la tumeur, entraînant un hématome qui risque de causer une complication neurologique, parfois facile à éviter. Depuis que l'on a recours à la vertébroplastie pour renforcer le “pilier antérieur” de la vertèbre [7], les montages courts (1 +1) sont de plus en plus fréquents. La technique chirurgicale pour les tumeurs rachidiennes est bien décrite dans l'Encyclopédie médico-chirurgicale [4].

**Figure 5:**
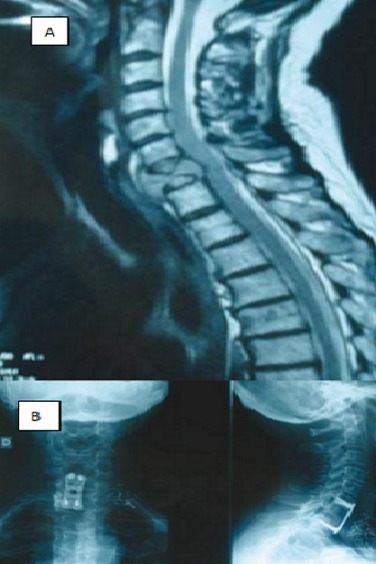
Métastase C7 d’un adénocarcinome de la prostate: Corporectomie de C7 par voie antérieure et Arthrodèse cervicale antérieure C6T1 par voie pré-sterno-cleïdo-mastoïdienne

**Métastases du rachis cervical:** Le risque de tétraplégie est rare, car le canal vertébral est large à ce niveau. La voie d'abord dépend de la localisation tumorale. En cas d'envahissement corporéal, la voie antérieure pré-sterno-cléido-mastoïdienne gauche plutôt que droite, à cause du nerf récurrent, est conseillée. Le risque de développer une infection est moindre avec cette voie d'abord comparée à un abord par voie postérieure, mais elle est plus technique. Une corporectomie avec une reconstruction par cage et plaque est le plus souvent utilisée. En cas d'envahissement tumoral très important s'étendant à l'artère vertébrale ou, cas plus rare, à toute la carotide, il ne faut pas hésiter à recourir à un spécialiste de la chirurgie vasculaire. Si l'atteinte est antérieure avec un envahissement canalaire, on peut choisir, selon les cas, qui doivent être discutés en réunion de concertation pluridisciplinaire (RCP), la voie antérieure ou la voie postérieure. En cas d'envahissement postérieur, la voie postérieure est la seule possible. Une laminectomie avec fixation est le plus souvent effectuée. Il faut pratiquer la résection de l'épidurite en faisant attention à ne pas créer de brèche pouvant entraîner une méningite carcinologique. On n'hésitera pas à étendre le montage si plusieurs vertèbres sont envahies (Figure 5).

**Métastases du rachis dorsal:** Cette localisation anatomique est la plus pourvoyeuse de complications neurologiques, car le diamètre du canal rachidien est étroit. La plupart du temps, on utilise la voie postérieure pour une laminectomie-fixation. La voie antérieure par thoracotomie est lourde techniquement et n'est recommandée que dans de rares cas. Là aussi, il ne faut pas hésiter à demander l'aide d'un spécialiste de la chirurgie thoracique si vous n'êtes pas entraîné à ce type d'abord. En cas d'atteinte tumorale importante, une voie postérolatérale élargie peut être empruntée. Il faut, au préalable, avoir fait une artériographie avec ou sans embolisation pour bien situer l'artère d'Adamkiewicz (artère nourricière médullaire). Une chirurgie carcinologique est encore possible si un seul des pédicules [1] est atteint, en ayant recours à la voie postérolatérale élargie.

**Figure 6:**
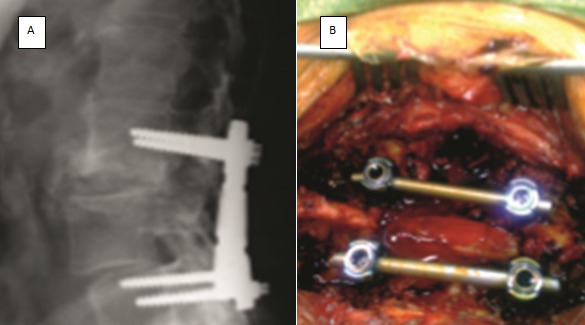
A) arthrodèse lombaire par plaques et vis pédiculaires pour métastase lombaire); B) vue peropératoire d’une résection tumorale droite, libération du fourreau dural et fixation par tige et vis pédiculaires

**Métastases lombaires:** Le risque de complication neurologique est peu élevé en raison de leur localisation anatomique, la moelle se terminant en D12-L1 le plus souvent. La voie d'abord peut être postérieure ou antérieure, selon les habitudes des équipes et la localisation de l'envahissement tumoral sur la vertèbre. Selon R. Roy-Camille [1], si un seul des pédicules est envahi, cela empêche toute chirurgie carcinologique. La voie postérolatérale est impossible à ce niveau à cause de la profondeur anatomique, interdisant le contrôle de la face antérieure de la vertèbre. La chirurgie consiste le plus souvent en une laminectomie-fixation. Elle est plus aisée, car, en dessous de L2, en général, on peut délicatement mobiliser le fourreau dural pour réséquer la tumeur. (Figure 6).

**Métastases sacrées:** Cette localisation anatomique ne permet pas de fixation. Seule une libération canalaire est possible, parfois associée à une vertébrectomie par voie postérieure en curetant la tumeur. Cela nécessite une bonne préparation du patient par le biais d'une embolisation préopératoire sur ces métastases, qui sont, en général, de volume important.

La dificulté de cette chirurgie est de prendre la bonne décision au bon moment pour atteindre le but choisi sans perdre de temps pour le traitement général de la maladie primitive. Certains auteurs ont essayé d'aider au mieux les chirurgiens à faire le bon choix en leur proposant un schéma thérapeutique selon la localisation tumorale. F. Denis [6] a été l'un des pionniers avec sa théorie des “3 piliers” en modélisant la vertèbre pour prédire le risque fracturaire. K. Tomita et al. [7] proposent une stratégie chirurgicale fondée sur la résection en bloc de la métastase rachidienne, prouvant que la survie est plus longue que celle des patients qui ont subi une résection intralésionnelle. Malheureusement, les métastases uniques sont extrêmement rares. Ces indications chirurgicales sont réservées à des cas très particuliers. S. Boriani et al. [8] décrivent une stratégie chirurgicale selon la localisation de la tumeur sur la vertèbre en détaillant bien les différentes techniques adéquates. Mais les indications sont réservées aux tumeurs primitives et non aux métastases, ce qui est bien différent. Même si une métastase est unique à un instant, rien ne permet de prédire qu'une autre métastase ne va pas apparaître ailleurs sur le squelette après plus ou moins longtemps. Cela est surtout avéré en cas de métastases d'origine mammaire. Trop souvent, on voit apparaître une métastase unique bien des années après le cancer primitif ; on décide donc une chirurgie agressive pour avoir une masse tumorale à 0 et, malheureusement, quelques mois après cette chirurgie, on découvre une autre métastase. On peut réaliser une superbe chirurgie en bloc et déplorer le décès du patient quelques mois plus tard. Il aurait peut-être fallu faire une chirurgie moins agressive en diminuant le temps postopératoire en hospitalisation, pour permettre au patient de profiter des jours de survie restant au sein de sa famille. Il faut se poser la question en permanence avant de prendre une décision chirurgicale en RCP. Concernant les métastases rachidiennes, Y. Tokuhashi et al. [2] ont été les premiers à essayer d'intégrer la métastase dans le bilan général de la maladie cancéreuse en proposant un arbre décisionnel. J. Feliu et al. [9] ont développé et validé un nomogramme pronostique pour les patients en phase terminale de maladies cancéreuses en se fondant sur les taux de LDH, de lymphocytes, d'albumine, et sur le délai entre le diagnostic initial et le stade terminal. Avec leur étude, ils nous renseignent sur un laps de temps de survie. C'est très novateur, car ils font partie des premiers à s'intéresser au temps de survie du patient cancéreux selon des critères objectifs. Mais cette méthode n'est applicable qu'aux patients en phase terminale, qui ne sont, a priori, plus opérables.

## Conclusion

Nous voyons que l'idéal pour la stratégie chirurgicale est d'avoir un arbre décisionnel regroupant les techniques chirurgicales selon l'anatomie de la métastase, en tenant compte du stade de la maladie cancéreuse, donc de la survie du patient. Cela nous permettrait de prendre la bonne décision au bon moment. Il ne faut pas oublier que la chirurgie des métastases osseuses est le plus souvent palliative, en raison de leur multiplicité. Elle doit être la plus adaptée au but choisi, tout en étant la plus simple possible pour éviter une hospitalisation longue, et s'insérer dans le traitement général de la maladie sans le retarder. C'est pourquoi toute décision chirurgicale doit être prise lors d'une RCP regroupant toutes les techniques médicales, radio- interventionnelles et chirurgicales. Ainsi, la chirurgie des métastases osseuses ne guérit ou n'augmente la durée de survie que rarement, mais améliore le plus souvent la qualité de vie du patient. Le cancer de la prostate est l'un des grands pourvoyeurs de métastases osseuses. Le piège est de croire, comme nous l'avons dit en préambule, à la solidité de la métastase condensante. Les fractures sont fréquentes, avec des conséquences neurologiques, surtout au niveau dorsal haut. Trop souvent, les équipes soignantes savent depuis longtemps que ces métastases sont présentes mais n'en estiment pas le risque réel. Chaque localisation métastatique doit être discutée en RCP pour éviter les complications fracturaires et neurologiques. Dès qu'une métastase présente un risque, une chirurgie doit être préconisée avant la radiothérapie, si l'état du patient le permet. En effet, la radiothérapie augmente les risques d'infections nosocomiales de 50 % en postopératoire et entraîne des diffcultés peropératoires en augmentant la fibrose des parties molles. Cette radiothérapie sera de toute façon effectuée 3 semaines après l'intervention. La chirurgie des métastases est le plus souvent palliative. Elle doit être rapide et sans complications, pour que le traitement adjuvant soit repris dès que possible.

### Etat des connaissances actuelles sur le sujet

- La chirurgie de la métastase est une chirurgie palliative le plus souvent, surtout au niveau du rachis de par sa localisation anatomique
- Le principal risque de la localisation rachidienne est neurologique, c'est pourquoi cette chirurgie doit être préventive le plus souvent
- Elle a pour objectif principal d'améliorer la qualité de vie.

### Contribution de notre étude à la connaissance

- la stratégie chirurgicale doit prendre en compte des paramètres primordiaux tels que le cancer primitif, l'objectif recherché, le stade de la maladie, l'état général du patient;
- Une réunion multidisciplinaire est donc primordiale avant toute décision chirurgicale.
